# Visualizing subatomic orbital and spin moments using a scanning transmission electron microscope

**DOI:** 10.1038/s41563-025-02242-6

**Published:** 2025-05-12

**Authors:** Hasan Ali, Jan Rusz, Daniel E. Bürgler, Joseph V. Vas, Lei Jin, Roman Adam, Claus M. Schneider, Rafal E. Dunin-Borkowski

**Affiliations:** 1 Department of Materials Science and Engineering, Uppsala University, Uppsala, Sweden; 2 Ernst Ruska-Centre for Microscopy and Spectroscopy with Electrons, Forschungszentrum Jülich, Jülich, Germany; 3 Department of Physics and Astronomy, Uppsala University, Uppsala, Sweden; 4 Peter Grünberg Institute, Forschungszentrum Jülich, Jülich, Germany

**Keywords:** Microscopy, Magnetic properties and materials, Ferromagnetism

## Abstract

Magnetism originates from the spin and orbital angular momenta of electrons and their coupling. These interactions occur at subatomic scales and a comprehensive understanding of such phenomena relies on characterization techniques capable of probing the spin and orbital moments at atomic resolution. Although electron energy loss magnetic chiral dichroism has previously enabled the detection of magnetic moments at atomic scales, it was limited to a chromatic-aberration-corrected transmission electron microscope. Although possible, the detection of atomic-scale electron energy loss magnetic chiral dichroism in a scanning transmission electron microscope remains elusive due to challenges associated with convergent beam setups. Here we demonstrate the detection of atomic-scale electron energy loss magnetic chiral dichroism signals in a probe-corrected scanning transmission electron microscope. We not only determine the orbital-to-spin moments ratio for individual atomic planes of an iron crystal but also reveal its local variations at subatomic scales. These findings open the possibility of resolving magnetism down to the orbital level in future studies.

## Abstract (web summary)

The orbital-to-spin moments ratio for individual atomic planes of an iron crystal is studied, revealing local variations at subatomic scales.

## Main

A deep understanding of quantum mechanical phenomena controlling magnetism such as spin–orbit coupling^1,2^, spin-splitting^3^ and spin^4,5^ or orbital Hall effects^6^ is crucial for the development of next-generation magnetic and spintronics devices. To detect and manipulate these effects, advanced characterization techniques are required that can probe magnetic behaviours at atomic or even subatomic scales. However, many commonly used magnetic characterization techniques such as scanning tunnelling microscopy^7^, magnetic force microscopy^8^, X-ray magnetic circular dichroism^9^ and electron holography^10^ are either surface sensitive or have a limited spatial resolution. Recent advancements in differential phase contrast and electron holography have shown promise in achieving magnetic measurements at the atomic resolution^11–13^. Nevertheless, these approaches either require specialized equipment^11,12^ or are limited to specific antiferromagnetic materials^13^. Moreover, in differential phase contrast and holography experiments, it is challenging to completely remove electrostatic contributions from the magnetic signal. Additionally, these methods lack the ability to resolve the orbital and spin contributions to the overall magnetic behaviour in a material.

A possibility to resolve the element-specific orbital and spin configurations at atomic spatial resolution was opened with the discovery of electron energy loss magnetic chiral dichroism (EMCD)^14^, an electron analogue of X-ray magnetic circular dichroism. Unlike X-ray magnetic circular dichroism, which relies on polarized photons, EMCD uses the crystal lattice within a transmission electron microscope (TEM) to split the electron beam, producing dichroic effects at conjugate scattering angles in the diffraction plane. The difference in the electron energy loss (EELS) spectra acquired at these scattering angles produces the EMCD signal, which can be analysed to determine the element-specific orbital and spin moments by applying theoretical sum rules^15,16^. Unlike X-rays, electrons—due to their charged nature—can be strongly focused to produce atomic-sized probes^17^, thereby opening the possibility to map the magnetic moments with atomic resolution.

**Extended Data Fig. 1:**
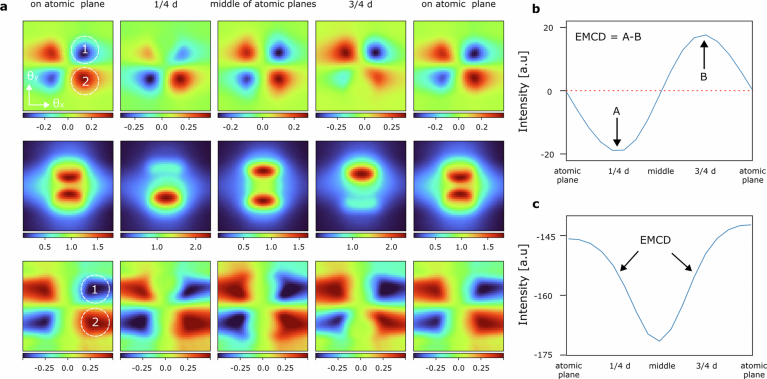
A comparison of STEM-EMCD and beam-shift EMCD. **a**, Simulated distribution of magnetic (top), non-magnetic (middle) and relative magnetic (bottom) signals in the reciprocal space as a function of probe position between two (110) atomic planes of Fe. The maps cover a scattering range of ±25 mrad (51 mrad) along both θ_x_ and θ_y_ dimensions. The plus and minus chiral signals (represented by red and blue colors) are symmetric on and in the middle of atomic planes and asymmetric at ±1/4 d positions where d is the atomic plane spacing (2 Å in this case). **b**, Beam-shift EMCD signal (a sum of detector positions 1 and 2) plotted as a function of probe-position, EMCD signal is obtained by taking the difference of intensities at ¼ and ¾ (-¼) positions. **c**, STEM-EMCD signal (difference of detector positions 1 and 2) plotted as a function of probe position, EMCD signal is detectable at all spatial positions on and between the two atomic planes and the strength of the signal is about an order of magnitude higher than beam-shift EMCD.

Classically, the EMCD experiments are conducted by tilting the crystal to a two- or three-beam orientation and recording two or four momentum-resolved EELS spectra^18^. In such configurations, although the atomic column resolution is lost, atomic planes can still be resolved. Despite the inherently non-local nature of inelastic electron scattering, EMCD has been found to strongly localize to atomic planes^19^, making it well suited for studying the evolution of magnetic properties at the atomic plane resolution. Most recently, EMCD signals with atomic plane resolution have been detected under parallel illumination conditions^20^. However, these experiments are limited to a chromatic-aberration-corrected (C_C_) TEM. Moreover, two sequential acquisitions are required in these experiments, complicating precise spatial registration between the two datasets due to specimen drift. A simpler and potentially more effective approach is to use an atomic-sized electron probe in a scanning TEM (STEM) instrument to map the EMCD signals^21^ at atomic resolution. However, the electron beam must be strongly converged to produce an atomically sharp probe and the strength of the EMCD signal decreases with an increasing convergence angle^21^. Additionally, as the diffraction discs in such setups start to overlap with the weaker EMCD signals, angular selection becomes challenging, making it difficult to isolate the EMCD signal. Moreover, classical STEM–EMCD experiments require the acquisition of two or more momentum-resolved EELS spectra, which poses additional challenges, particularly in controlling specimen drift to ensure the precise spatial registration of multiple datasets. These complications have led to the assumption that detecting atomically resolved EMCD signals in classical STEM setups is hard to achieve. Instead, an alternative experimental geometry called beam-shift EMCD was proposed, in which instead of taking the difference between the momentum-resolved EELS spectra, the EMCD signal is obtained by taking the difference in the EELS spectra acquired at precise conjugate displacement around an atomic plane^22^, allowing data to be collected in a single scan. A comparison of STEM–EMCD and beam-shift EMCD (Supplementary Note 1 and Extended Data Fig. 1) reveals that the signal strength in STEM–EMCD experiments is about an order of magnitude higher than beam-shift EMCD experiments. Thus, although STEM–EMCD is more challenging, it remains the superior method for mapping atomic-scale signals and gives far better and deeper insights into atomic-scale magnetism. In this paper, we present quantitative STEM–EMCD experiments performed on a 10-nm-thick iron (Fe) crystal. These experiments not only demonstrate the detection of EMCD signals from individual atomic planes but also reveal EMCD variations within interatomic spaces. We observe notable differences of orbital and spin contributions between atomic planes and interstitial regions. Density functional theory simulations of Fe slabs^23^ attribute these subatomic variations in magnetic properties to surface effects, which appear to dominate at the centre of atomic planes.

## Conceptual framework and experimental design

One of the primary challenges associated with EMCD experiments, especially when targeting atomic resolution, is the need to acquire multiple momentum-resolved EELS spectra by scanning the same sample region repeatedly^21^. Maintaining consistent experimental conditions across these subsequent scans is non-trivial due to factors such as beam damage, contamination and spatial drift. These issues have historically impeded achieving atomic resolution in classical STEM–EMCD experiments. To address this challenge, we conducted the EMCD experiments in the *q*–*E* mode^24^ in which the momentum resolution of non-overlapping EMCD signals along the *θ*_*y*_ axis in the diffraction plane is preserved when projected onto the energy loss axis. This approach enables the EMCD experiment to be performed in a single scan^25^. For further details about this setup, the reader is referred to our previous works^25–27^.

**Fig. 1:**
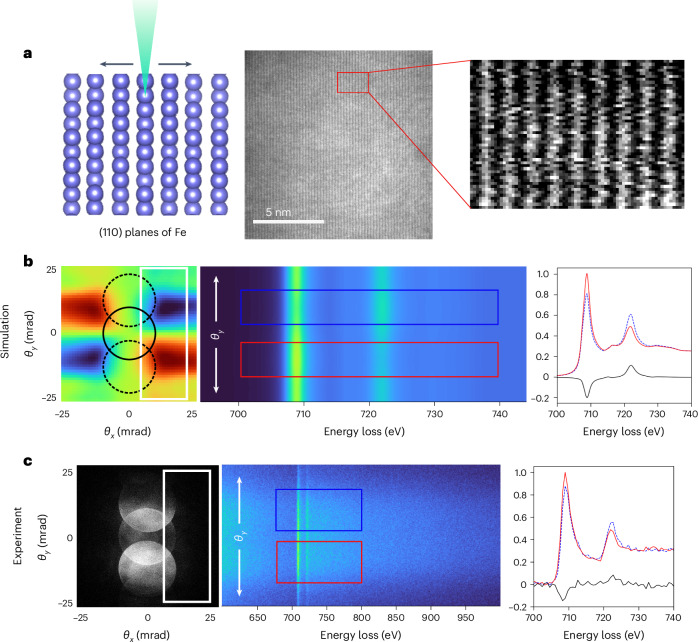
A conceptual depiction of the experimental setup. **a**, Schematic showing the (110) atomic planes of Fe oriented parallel to the electron beam (left), experimental HAADF image of an Fe crystal oriented along (110) atomic planes (middle) and HAADF image of the area mapped for EMCD measurements (right). **b**, Simulated EMCD signal distribution in the reciprocal space with (±110) diffraction discs excited for a convergence semiangle of 10 mrad and the position of the slit aperture is indicated by the white rectangle (left). The simulated 2D EELS image for the signal integrated in the slit; here the *θ*_*y*_ axis is preserved in such an image, whereas the *θ*_*x*_ axis is collapsed into the energy loss axis (middle). The simulated EELS spectra extracted from two chiral *θ*_*y*_ positions indicated by the blue and red rectangles and the corresponding EMCD signal (right). **c**, Experimental diffraction pattern with (±110) diffraction discs excited for a convergence semiangle of 10 mrad; the position of the slit aperture is indicated by the white rectangle (left). The experimental 2D EELS image for the signal integrated in the slit; this image is summed up for nine atomic planes (middle). The experimental raw EELS spectra extracted from the two chiral *θ*_*y*_ positions indicated by the blue and red rectangles and the corresponding EMCD signal (right).

Figure 1 illustrates the experimental setup used in this study. An Fe crystal is tilted to a three-beam orientation, aligning the (110) atomic plans parallel to the incoming electron beam (Fig. 1a). We first use simulated data to conceptualize the detection of the EMCD signal in a single scan under atomic plane resolution conditions followed by the experimental detection of EMCD under the same conditions. With the Fe crystal in a three-beam orientation, the simulated EMCD signal has four chiral components distributed in the diffraction plane (Fig. 1b, left). In this image, the solid circle represents the direct-beam position, whereas the dashed circles indicate the diffracted-beam positions (*g* = ±110). Of the four EMCD components, two are sufficient to extract a quantitative EMCD signal. By placing a slit aperture in the configuration marked by a white rectangle, two EMCD components with opposite chirality can be simultaneously projected onto the detector, forming a two-dimensional (2D) EELS image (also called a *q*–*E* image). Figure 1b (middle) shows the simulated 2D EELS image, where the blue and red rectangles indicate the regions used to extract the chiral EELS spectra. These spectra, along with their difference spectrum (EMCD signal), are shown in Fig. 1b (right).

The experimental results, obtained following the same methodology, are presented in Fig. 1c. The left panel shows the experimental diffraction pattern of Fe under a three-beam orientation (*g* = ±110), with the slit position marked by a white rectangle. The middle panel displays the experimental 2D EELS image, integrated over nine atomic planes. The chiral EELS spectra were extracted from the regions marked by the blue and red rectangles. These experimental (raw) spectra along with the EMCD signal are shown in Fig. 1c (right). A clear EMCD signal is detected, confirming the validity of the experimental setup.

**Extended Data Fig. 2:**
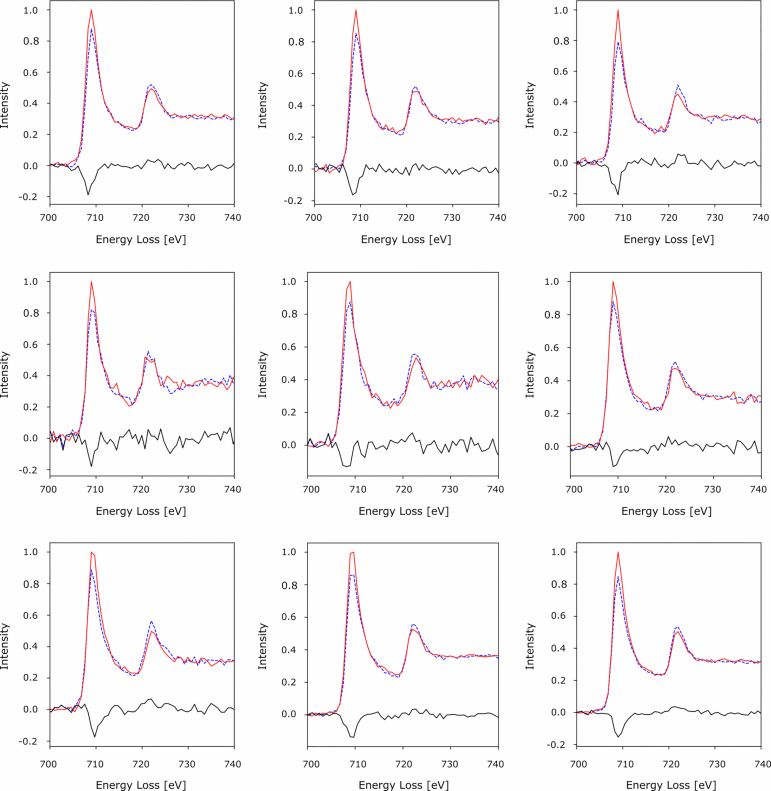
Atomic plane resolved EMCD signals from nine different datasets. Raw EELS spectra and EMCD signals extracted from nine different datasets excluding the two datasets shown in the paper. These datasets were acquired from different grains of Fe. These spectra were produced by integrating all pixels in each dataset. The EELS spectra were background-subtracted and post-edge normalized as described in the Methods section.

To assess reproducibility, we collected over ten datasets and observed an EMCD signal in all instances, demonstrating the robustness of this technique. The total (raw) EMCD signals extracted from nine additional datasets (excluding the two datasets presented in this paper) are presented in Extended Data Fig. 2. All the EELS spectra presented in Fig. 1 and Extended Data Fig. 2 were background subtracted and post-edge normalized (Methods).

## Quantitative EMCD signals from individual atomic planes

**Fig. 2:**
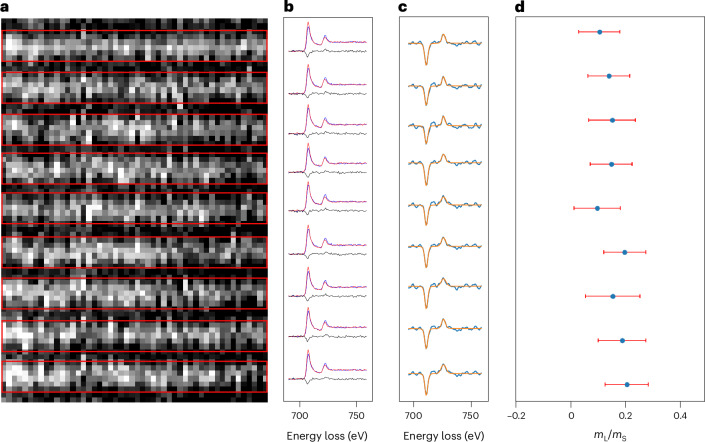
EMCD signals from individual atomic planes. **a**, HAADF image showing nine (110) atomic planes of Fe. The pairs of chiral EELS spectra for each atomic plane were extracted by integrating the spectra within areas marked by the red rectangles. **b**, Pair of chiral EELS spectra and the corresponding EMCD signal for each atomic plane. The EELS spectra shown here have been background subtracted and post-edge normalized (Methods). **c**, Gaussian-filtered EMCD signals fitted with pseudo-Voigt curves. **d**, *m*_L_/*m*_S_ determined for each atomic plane. The nominal *m*_L_/*m*_S_ values shown here were determined by applying sum rules to the fitted EMCD signal obtained for each atomic plane. The error bars represent the combined uncertainty from two sources, estimated in quadrature: the uncertainty of the curve-fitting parameters and the statistical (random) error derived from the residuals between the original and fitted EMCD signals. A detailed description of error analysis is given in Supplementary Note 3.

**Extended Data Fig. 3:**
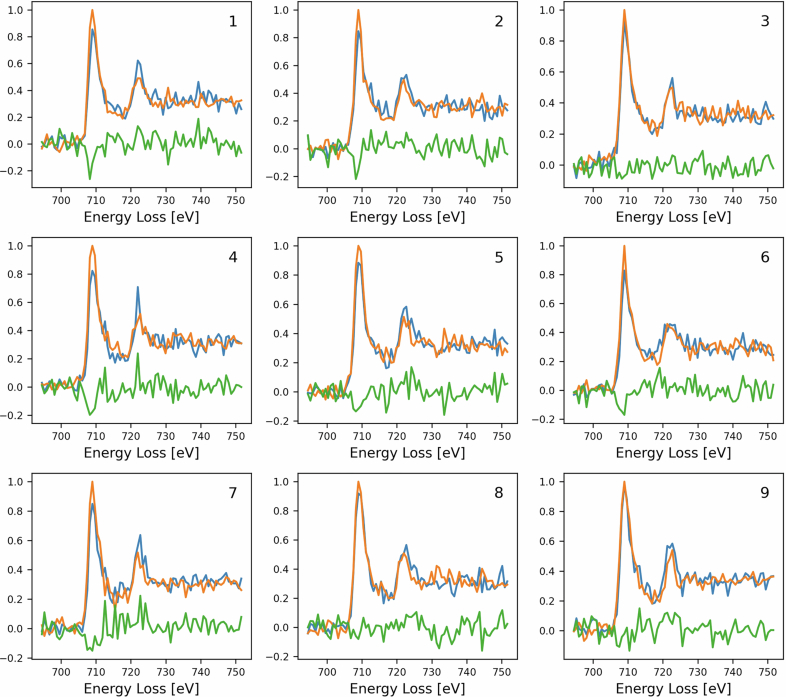
Raw EMCD signals extracted from individual atomic planes. Raw EELS spectra and EMCD signals extracted from nine individual atomic planes for the data shown in Fig. 2. The EELS spectra were background-subtracted and post-edge normalized as described in Methods section.

To obtain the EMCD signals from individual atomic planes, we integrated the 2D EELS images for each atomic plane. The integration areas corresponding to each atomic plane are indicated (Fig. 2a, red rectangles). For each atomic plane, a pair of EELS spectra were extracted from the corresponding 2D EELS image within a scattering angle ranging from 2 to 18 mrad and –2 to –18 mrad. The background of each spectrum was subtracted, and the post-edge normalization was applied following the procedure described in the Methods. The EMCD signal for each atomic plane was obtained by taking the difference between the corresponding pair of chiral EELS spectra. To reduce noise, robust principal component analysis was applied (Methods). A clear EMCD signal was observed at each atomic plane (Fig. 2b). For quantification, each EMCD signal was filtered using a Gaussian filter and was fitted with a pseudo-Voigt^28,29^ curve (Fig. 2c). The magnetic orbital-to-spin moments ratio (*m*_L_/*m*_S_) was determined by applying the sum rules^15,16^. A description about the determination of error bars is provided in Supplementary Note 3, and a comprehensive description of the data processing workflow is provided in the Methods. The raw EMCD signals for the nine atomic planes are shown in Extended Data Fig. 3.

**Extended Data Fig. 4:**
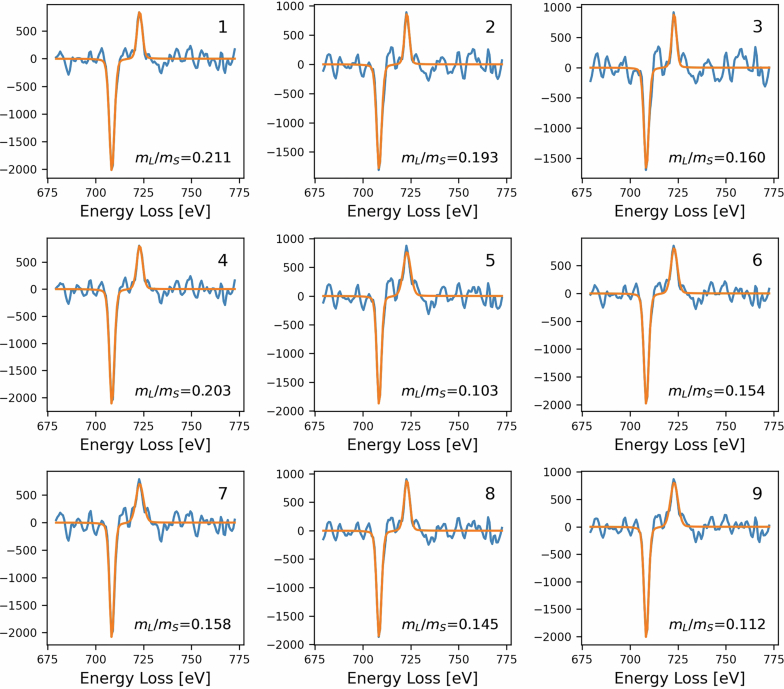
Curve-fitted EMCD signals from individual atomic planes. EMCD signals extracted from nine individual atomic planes shown in Fig. 2 fitted with pseudo-Voigt curves. Each EMCD signal is labelled with corresponding m_L_/m_S_ value determined by applying Sum rules to the fitted signal. The EMCD signals shown here were processed using robust PCA followed by a Gaussian filter as described in the Methods section.

Although the *m*_L_/*m*_S_ values obtained for individual atomic planes are similar within the error bars, they are higher than those previously reported for body-centred cubic (bcc) Fe (refs. ^30,31^), with an average *m*_L_/*m*_S_ value of 0.16 for the nine atomic planes. The curve-fitted EMCD signals for these nine atomic planes labelled with the corresponding *m*_L_/*m*_S_ values are presented in Extended Data Fig. 4. One common explanation for higher values observed in EMCD experiments is plural scattering^32^ of the electron beam as it traverses the sample, which can—in principle—be removed by deconvolving the core-loss EELS spectra with low-loss spectra acquired from the same region. However, in our case, the sample is relatively thin (10 nm), suggesting that plural scattering may not be the primary contributor to these higher values. In fact, for such thin samples, surface effects can dominate and strongly influence the physical properties of the overall system. Numerous studies have reported a substantial increase in the orbital magnetic moment for surface atoms^33–35^, specifically for Fe with reduced dimensionality^23,36–38^. In our measurements, the electron beam passing through the specimen results in a convolution of magnetic moments of both surface and bulk atoms, leading to a relatively higher total orbital contribution compared with bulk values.

## Interatomic variation of *m*_L_/*m*_S_

**Fig. 3:**
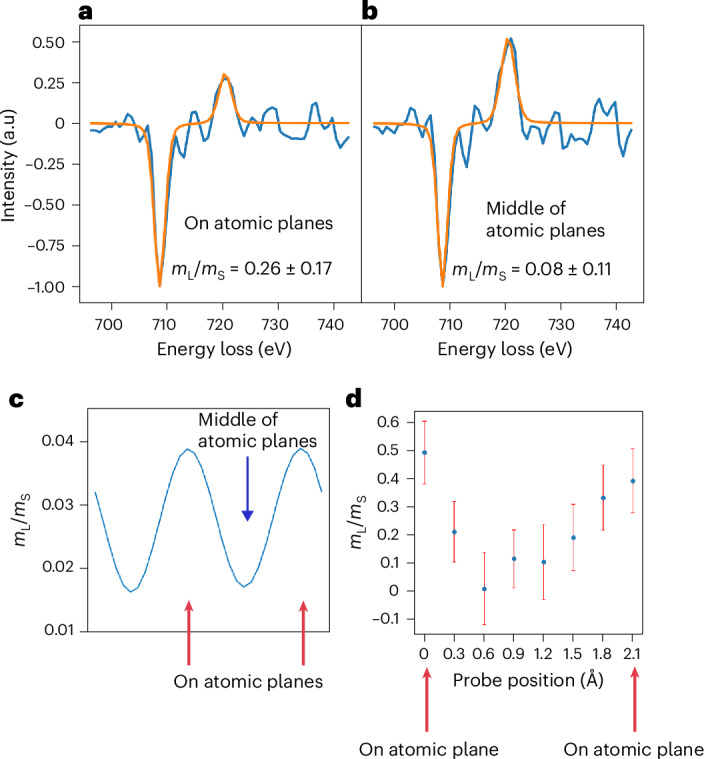
EMCD detection at subatomic plane resolution. **a**,**b**, EMCD signals with fitted curves integrated on (**a**) and between (**b**) nine atomic planes. These signals were acquired with a beam convergence semiangle of 15 mrad. There is an appreciable change in the white line ratio (L_3_/L_2_ edge intensities) and the resulting *m*_L_/*m*_S_ values for the two EMCD signals. **c**, Simulated *m*_L_/*m*_S_ values as a function of the probe position across the (110) atomic planes of Fe. The *m*_L_/*m*_S_ values for the surface layers^23^ have been included in these calculations; the *m*_L_/*m*_S_ ratio on and between the atomic planes varies systematically, with the values going lower between the atomic planes. **d**, Experimental *m*_L_/*m*_S_ values as a function of the probe position on and between two (110) atomic planes of Fe. The variation profile between the two atomic planes matches the trend seen in the simulated profile in **c**, although the modulation amplitude in the experiment is about an order of magnitude higher than the simulation. The *m*_L_/*m*_S_ values presented here are the nominal values obtained by applying the sum rules to the fitted EMCD signal obtained for each probe position. The error bars reflect the combined uncertainty from two sources, estimated in quadrature: the uncertainty of the curve-fitting parameters and the statistical (random) error derived from the residuals between the original and fitted EMCD signals. A detailed description of the error analysis is provided in Supplementary Note 3.

A key advantage of the experimental setup presented here, compared with the previously reported beam-shift EMCD method, is its ability to extract the EMCD signal at any spatial point between two adjacent atomic planes (Supplementary Note 1 and Extended Data Fig. 1). This capability allows us to analyse the EMCD signal as a function of the probe position, enabling subatomic magnetic measurements. To achieve a more localized signal, we increased the convergence semiangle of the electron probe to 15 mrad and conducted the experiment under conditions similar to those illustrated in Fig. 1. The EMCD signals averaged over atomic planes and interplanar regions together with their measured *m*_L_/*m*_S_ ratios are presented in Fig. 3a,b. It is important to note that the *m*_L_/*m*_S_ ratio of an EMCD signal is strongly influenced by the white line ratio (that is, the L_3_/L_2_ edge intensity ratio). For a direct comparison, the maximum amplitude of the L_3_ peak in both signals was normalized to –1. Although large error bars reduce confidence in statistical significance of the difference in the measured *m*_L_/*m*_S_ values for the two EMCD signals, the clear variation in the white line ratio between the two signals strongly indicates a change in the magnetic properties on and between the atomic planes. The measured *m*_L_/*m*_S_ value between the atomic planes is lower and closer to the standard values reported for bcc Fe (ref. ^31^).

**Extended Data Fig. 5:**
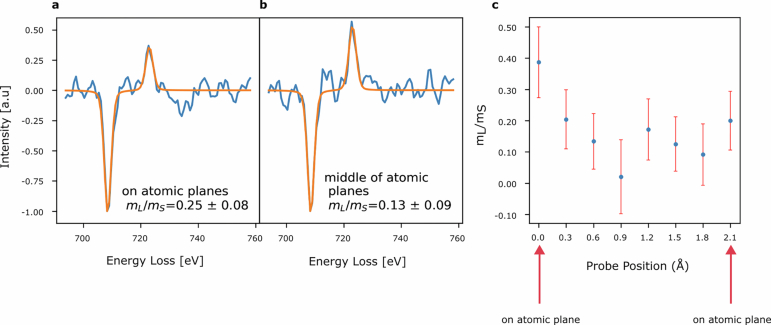
Comparison of EMCD signals on and between (110) atomic planes of Fe. **a-b**, EMCD signals on and between (110) atomic planes of Fe respectively for the data acquired with 10 mrad convergence semi-angle. These EMCD signals have been averaged over and between nine atomic planes. A variation in white line ratio between the two EMCD signals indicates a change in magnetic properties which is also evident from the measured m_L_/m_S_ ratios. **c**, m_L_/m_S_ values as a function of probe-position between adjacent atomic planes, EMCD signal for each probe position was averaged over nine atomic planes (see Methods for details).

Although it is difficult to rule out the effect of non-dipole transitions, consistently collecting the data in the same range of scattering angles makes this explanation rather unlikely. Instead, we believe that we are observing varying contributions from atomic layers across the sample thickness. When the electron beam is positioned directly over an atomic plane, surface atoms have a more pronounced impact on the observed spectra. Conversely, a beam positioned between the planes travels through the sample for a certain distance until its average distance from the Fe atoms decreases and, thus, it is mostly bulk atoms that will contribute to the EELS signal. We simulated the atomic-probe-dependent *m*_L_/*m*_S_ ratios for a 10-nm bcc Fe incorporating values for the surface layers referenced here^23^. The results indicate a non-negligible change in *m*_L_/*m*_S_ values on and between the atomic planes (Fig. 3c). Following the simulation, we extracted experimental EMCD signals at eight probe positions between two atomic planes and observed a systematic variation in the measured *m*_L_/*m*_S_ values, showing a similar trend seen in the simulations. This supports our hypothesis that the dominance of surface effects at the atomic centres contributes to these variations. Although the variations observed between the two atomic planes are driven by surface effects, this finding underscores the unprecedented subatomic sensitivity of our measurements. We also performed probe-dependent measurements for the data presented in Fig. 2, acquired with a beam convergence semiangle of 10 mrad. These data also exhibit a similar trend as shown in Extended Data Fig. 5, although the observed variation is less pronounced due to higher delocalization effects resulting from a larger probe size.

## Discussion and outlook

Due to its applicability to map the magnetic moments under magnetic-field conditions, STEM–EMCD is a favourable technique to obtain atomic-resolution magnetic measurements in any probe-corrected STEM equipped with an EELS spectrometer. The resolution achieved in these experiments enables the tracking of subatomic magnetic variations in other physically interesting systems. For instance, examining the variations in orbital and spin moments between two antiferromagnetically coupled planes could potentially enhance our fundamental understanding of the magnetic behaviour in such systems. Recently, STEM–EELS has been utilized for the spatial mapping of orbitals^39,40^. In our experiments, we have achieved the sensitivity required for such measurements. In the future, there can be possibilities to map the magnetic moments with orbital spatial resolution.

The changes in *m*_L_/*m*_S_ ratios on and between the atomic planes observed in the experimental data are approximately an order of magnitude higher than those predicted by simulations (Fig. 3). Beyond surface effects, other factors may contribute to this subatomic variation in *m*_L_/*m*_S_ ratios. As mentioned earlier, plural scattering can lead to larger values of *m*_L_/*m*_S_ and it is plausible that the plural scattering varies non-uniformly between atomic centres and interatomic spaces. This can be investigated as a possible extension of this study in the future. Additionally, there may be other quantum mechanical phenomena at play, such as orbital overlap or changes in the angular momentum of electrons as a function of displacement from the nucleus, which could also contribute to these variations. However, exploring these possibilities is beyond the scope of this work, leaving them as open questions for future research.

## Methods

### Sample fabrication

The sample was prepared by the thermal vapour deposition of 10-nm Fe onto a 5-nm-thick Si_3_N_4_ membrane (SiMPore), which was at room temperature. The average lateral grain size of the polycrystalline bcc Fe film was increased to about 50 nm by subsequent annealing in a vacuum at 1,050 K for 2 h (ref. ^22^). A 2-nm-thick Al cap layer deposited at room temperature by evaporation protects the Fe film from oxidation on air exposure, which was confirmed by the absence of Fe oxide signals in the EELS spectra (this analysis is presented in Supplementary Note 2). The Fe and Al thicknesses were controlled by calibrated quartz microbalances.

### Simulations

First, a minimal orthogonal Fe supercell with the *c* axis parallel to the [118] direction has been constructed. It contains 132 Fe atoms and its dimensions are √33*a* × √2*a* × √66*a*, where *a* = 2.87 Å is the lattice parameter of bcc Fe. This structure model was then periodically repeated 3 × 12 × 6 times along the *x*, *y* and *z* directions, respectively, to reach sufficient lateral dimensions preventing convergent probe overlaps with its periodic copies as well as to reach the target sample thickness of around 10 nm. Simulations were done using the MATS v. 2 method^41^, with the convergence parameter set to 0.00001. For each beam position, an Fe-L_3_-filtered diffraction pattern was calculated within the range from −25 mrad to +25 mrad in both scattering directions, with a grid step of 1 mrad.

Spectral simulations shown in Fig. 1b originate from a previous work^42^ and simulations of the beam-position-dependent *m*_L_/*m*_S_ ratio are based on the values of spin and orbital magnetic moments reported in ref. ^23^, assigned to individual Fe atoms in the structure model described above.

### Data acquisition

The EMCD experiments were carried out on a Thermo Fisher Titan G3 50-300 PICO fourth-generation TEM. The microscope is equipped with a Schottky-type high-brightness electron gun (FEI X-FEG), a monochromator, a C_S_ probe corrector (CEOS DCOR), a C_S_-C_C_ achro-aplanat image corrector (CEOS CCOR+), a post-column imaging energy filter (Gatan Quantum 966 ERS) and a Gatan K3 direct electron detector. The experiments were carried out in the scanning TEM mode using an acceleration voltage of 300 kV and the probe corrector was tuned to get the atomic-resolution conditions at a beam convergence semiangle of 10 mrad. A suitable grain of Fe oriented to the three-beam condition with the (110) planes parallel to the electron beam was found. The selection of the grain was made following the criterion that the systematic row of reflections is as parallel to the slit aperture as possible. The residual rotation offset was compensated by rotating the diffraction pattern using the projector lens, ensuring that the diffraction pattern is perfectly parallel to the slit aperture. The diffraction pattern was aligned to the slit aperture in the way shown in Fig. 1c. An atomic-resolution high-angle annular dark-field (HAADF) image of the atomic planes was obtained and a small area shown by the red rectangle in Fig. 1a (middle) was assigned as a survey image for the acquisition of an EMCD dataset. A probe current of 60 pA was used and the step size was set to 0.3 Å. The slit aperture was inserted and four-dimensional (4D) STEM–EELS data were acquired, where a 2D EELS image was acquired at each beam position. The dwell time per pixel was set to 13 ms, resulting in a total acquisition time of 52 s. More than ten datasets were acquired from three different grains in the same session. The experiment with a 15-mrad convergence semiangle was performed on another day under the same experimental conditions, except that the probe current was 80 pA.

### Data analysis

The 4D STEM–EELS data were processed in Python using HyperSpy^43^ and other built-in libraries. First, the slight spatial drift across the atomic planes in the HAADF image was corrected by vertically aligining the maxima of the atomic planes. The same drift correction was applied to the 4D STEM–EELS data. The scaling factor for the *θ*_*y*_ axis between the 2D EELS images and diffraction pattern was determined (size of *θ*_*y*_ in the 2D EELS image/size of *θ*_*y*_ in the diffraction pattern = 512 pixels/3,456 pixels = 0.148). The *y* axis of the 2D EELS images was calibrated in mrad (*θ*_*y*_), whereas the *x* axis was calibrated in energy loss (eV) units. The details about these calibrations are provided in the data processing workflow in the Zenodo repository (ref. ^44^). From the 2D EELS images at each pixel of the 4D STEM–EELS data, two EELS spectra were extracted in the range from 2 mrad to 18 mrad and –2 mrad to –18 mrad and were put back into two empty three-dimensional datasets with the spatial dimensions equal to the original 4D STEM–EELS data. This produces two EELS spectrum images (SIs), here called chiral plus (2 mrad to 18 mrad) and chiral minus (−2 mrad to −18 mrad) SIs. The X-ray spikes were removed from both SIs. To reduce noise, a robust principal component analysis was applied to both SIs using the HyperSpy toolbox. Given that the EELS spectra exhibit Poissonian noise characteristics, the Poissonian noise was normalized. The Poisson noise normalization is available as a built-in feature and applying robust principal component analysis in HyperSpy, and the normalization is carried out using the procedure described in ref. ^45^. The screen plot suggested one significant component but to retain maximum variance in the data for probe-dependent EMCD analysis, the dataset was reconstructed using ten components. The chiral-plus and chiral-minus EELS spectra were extracted from both robust principal-component-analysis-processed SIs by integrating 6 × 50 pixels along each atomic plane in which the length of each plane is 50 pixels. The background of the EELS spectra was removed using a power-law model fitted in an energy interval of 650–700 eV. Then, the post-edges of each pair of the EELS spectra were normalized. For post-edge normalization, a 40-eV energy window (740–780 eV) was used. The difference in each pair of the spectra was taken to produce the EMCD signal. Each EMCD signal was filtered using a Gaussian filter to suppress high-frequency noise fluctuations and was fitted with a pseudo-Voigt function.The *m*_L_/*m*_S_ value for each EMCD signal was determined by applying sum rules using the method described in our recent work^25^. The same procedure was also applied to raw SIs to extract raw EMCD signals from each atomic plane (Extended Data Fig. 3).

For subatomic measurements, eight EELS spectra were extracted from the chiral-plus and chiral-minus SIs by integrating 1 × 50 pixels starting from the centre of the first atomic plane and offsetting by 1 pixel along the *x* axis for each next spectrum. The same procedure was repeated for all the nine atomic planes and the nine EELS spectra for each probe position were integrated to improve the signal-to-noise ratio, resulting in eight probe-dependent pairs of EELS spectra. These eight spectral pairs were processed to obtain the EMCD signals in the same way as described above. The eight probe-dependent EMCD signals were curve fitted and the *m*_L_/*m*_S_ value for each was determined. To determine the error bars, the uncertainty of the curve-fitting parameters and the random error were taken into account (details are given in Supplementary Note 3). We have posted the data processing workflow code and the raw data online in the Zenodo repository (ref. ^44^) to simplify testing and reproducing our results.

## Online content

Any methods, additional references, Nature Portfolio reporting summaries, source data, extended data, supplementary information, acknowledgements, peer review information; details of author contributions and competing interests; and statements of data and code availability are available at 10.1038/s41563-025-02242-6.

## Supplementary information

Supplementary Information Supplementary Notes 1–3 and Fig. 1.

## Data Availability

The relevant data supporting the findings of this study are available via Zenodo at 10.5281/zenodo.14827898 (ref. ^44^) and from the corresponding author upon reasonable request.

## Code availability

The data analysis code is available via Zenodo at 10.5281/zenodo.14827898 (ref. ^44^) in the form of Jupyter notebooks.
