# Supplementary material for: Visualizing subatomic orbital and spin moments using a scanning transmission electron microscope
Source: Nat Mater. 2025 May 12;24(8):1215–20. doi: 10.1038/s41563-025-02242-6 (PMC12310538; doi:10.1038/s41563-025-02242-6)
Supplement: Supplementary file 1 — Supplementary Notes 1–3 and Fig. 1. [file 41563_2025_2242_MOESM1_ESM.pdf]

nature materials

Supplementary information https://doi.org/10.1038/s41563-025-02242-6

Visualizing subatomic orbital and spin moments using a scanning transmission electron microscope

In the format provided by the authors and unedited

Contents

Supplementary Note 1. Comparison of STEM-EMCD and beam-shift EMCD ---------------- 2

Supplementary Note 2. Confirmation of non-oxidation of Fe ----------------------------------- 3

Supplementary Note 3. Error determination in quantification of m_L/m_S ratios ----------------- 5

S3.1. Curve Fitting Parameters' Uncertainty -- 5

S3.2. Random Error --- 5

S3.3. Total Error -- 5

Supplementary Note 1. Comparison of STEM-EMCD and beam-shift EMCD

For simplicity, we will call the EMCD experiments based on the difference of momentum-resolved EELS spectra as STEM-EMCD (used in this paper) and the EMCD experiments exploiting the signal around an atomic plane as beam-shift EMCD. Extended Data Fig. 1 (a) shows the simulated reciprocal space maps of magnetic, non-magnetic and relative magnetic (magnetic/non-magnetic) components of EMCD signal as a function of real space probe-position between two (110) atomic planes of Fe. In beam-shift EMCD experiments, a big detector is used to sum up the signals at positions 1 and 2, resulting in a net zero signal on and in the middle of the neighbouring atomic planes. However, at ¼ and ¾ displacements from the centre of the atomic plane, the asymmetric intensity variations of minus and plus components result in residual intensities on summation which are inverse to each other. A difference of intensities at these two positions is taken as the EMCD signal as shown in Extended Data Fig. 1 (b). Thus, the EMCD signal is dependent on the real space position of the probe instead of the reciprocal space, allowing to carry out the experiment in a single beam scan[1]. This also puts a stringent requirement to tightly control the real space position of the probe along an atomic plane as even an inadvertent offset by a single pixel could severely complicate the analysis. The STEM-EMCD experiments, on the other hand, solely depend on the angular selection of the intensities in reciprocal space where a difference of signals at detector positions 1 and 2 is taken as an EMCD signal as shown in Extended Data Fig. 1 (c). This not only gives access to EMCD signal at all the probe positions, but the strength of the signal is about an order of magnitude higher than the beam-shift EMCD. The challenge associated with STEM-EMCD experiments is the requirement to acquire at least two momentum-resolved signals which is typically done by sequentially scanning the region of interest[2], hindering the atomic resolution measurements. The experimental setup presented in this paper allows to acquire two momentum-resolved EELS spectra in a single scan, making the atomic resolution measurements possible.

Supplementary Note 2. Confirmation of non-oxidation of Fe

It is crucial to control the oxidation of Fe in these experiments as the oxidation can cause variations in white-line ratio (L_3/L_2 edge intensity ratio). To prevent oxidation of Fe, the Fe film was capped with a 2 nm Al layer. Moreover, the samples were stored in high vacuum after fabrication and transferred to TEM on the day of experiment, with a transport time of roughly 15 minutes. Nevertheless, Al immediately oxidizes to Al_2O_3 on exposure to air.

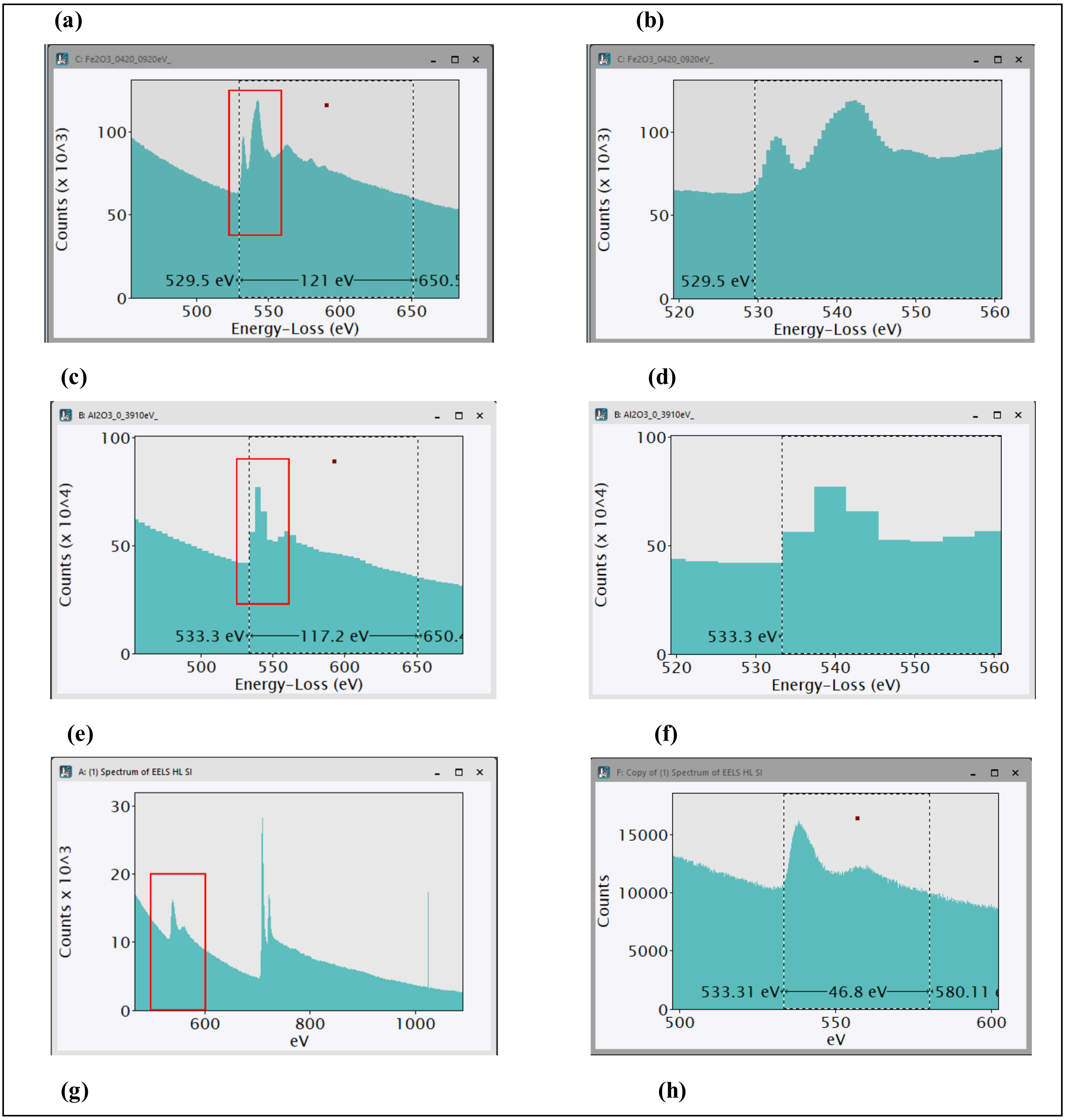

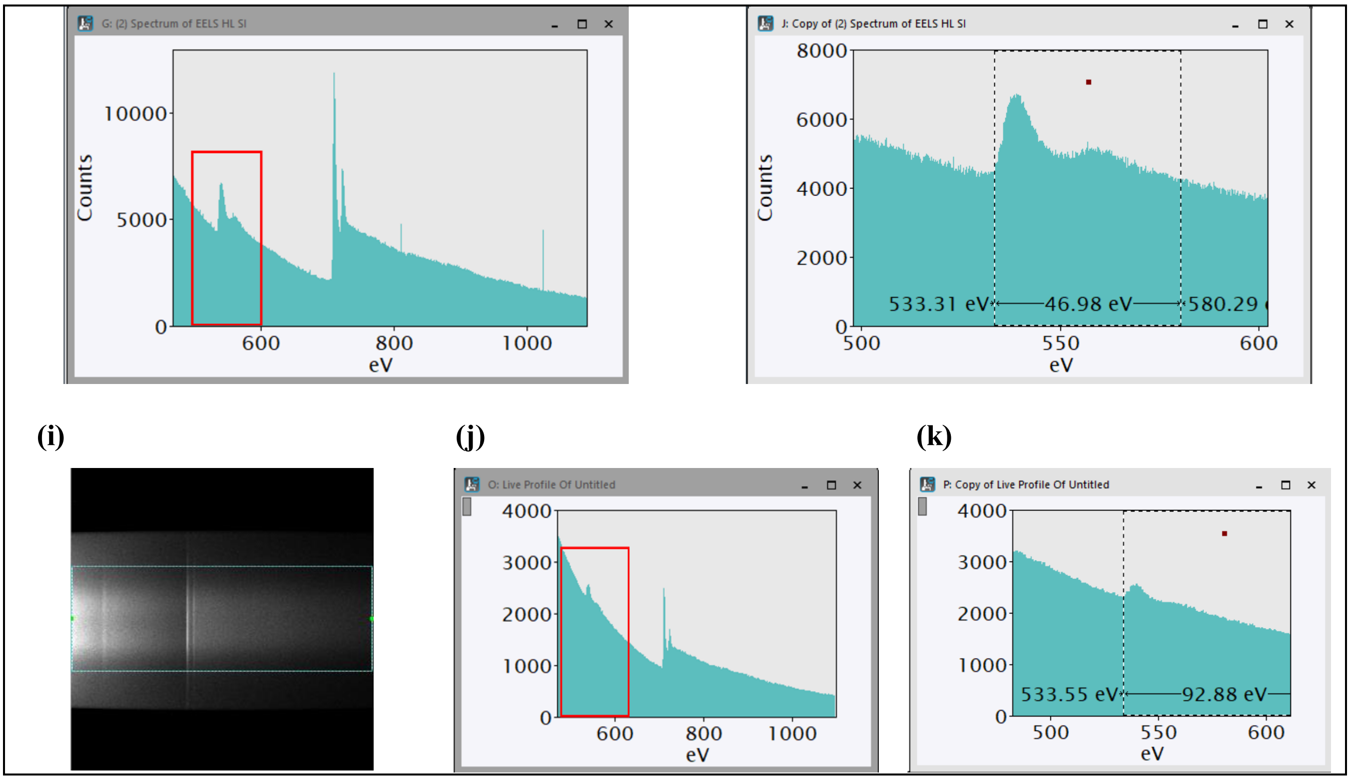

Supplementary Fig. 1. (a,b) reference O-K edge spectra for Fe_2O_3, (c,d) reference O-K edge spectra for Al_2O_3, (e,f) O-K edge spectra acquired before EMCD mapping, (g,h) O-K edge spectra acquired after EMCD mapping, (i,j,k) O-K edge spectra extracted from 4D data acquired during EMCD experiment. The red box in each spectrum indicates the region zoomed in for the next spectrum. The edge features and edge onset energy (~533.3 eV) of all the experimental spectra clearly match the Al_2O_3 reference spectrum.

To confirm that the Fe film was not oxidized, EELS spectra of Oxygen K-edge were acquired before, after and during the experiment as the Oxygen K-edge has distinct features and onset energy loss for Al_2O_3 and Fe_2O_3. The EELS spectrum images containing Oxygen K-edge before and after the EMCD experiment were acquired in dual-EELS format which means the zero-loss peak (ZLP) was acquired in parallel with the core-loss EELS and the resulting core-loss EELS spectra were accurately calibrated along energy-loss axis using ZLP as a reference. The data acquired during the EMCD experiment, however, was acquired in 4D-format due to EMCD experimental requirements and dual-EELS acquisition is currently not possible under these settings. The spectra in this case were calibrated considering the nominal energy loss values of Fe L_3 peak. The reference spectra for Al_2O_3 and Fe_2O_3 were taken from EELS Atlas [3]. Supplementary Fig. 1 shows the reference EELS spectra for Fe_2O_3 and Al_2O_3 and the EELS spectra containing Oxygen K-edge acquired before, after and during the EMCD experiment. All the experimentally acquired EELS spectra clearly match both in features as well as edge onset energy to the Al_2O_3 reference spectrum. We do not observe any peak corresponding to Fe_2O_3. Based on this analysis, we rule out any detectable levels of the oxidation of Fe.

Supplementary Note 3. Error determination in quantification of m_L/m_S ratios

To quantify the EMCD signals presented in this study, the signals were fitted with a pseudo-Voigt curve and the m_L/m_S ratio was determined by applying sum rules in a way described in our recent paper[4]. To estimate the analysis error, two error sources were considered as detailed below.

S 3.1. Curve Fitting Parameters' Uncertainty

In the curve-fitting function, four parameters (amplitudes and full-width half maxima of L_3 and L_2 edges) were kept as freely varying parameters. The uncertainty of these four parameters was determined in the following way. First, a Gaussian-filter (sigma=0.8-1.0) was applied to EMCD signal to reduce the effect of noise. Then the EMCD signal was fitted with a pseudo-Voigt function containing two inverse peaks. The co-variance matrix of the fitted parameters was obtained. The diagonal elements of the co-variance matrix represent the variance of fitted parameters whereas the uncertainty of each fitted parameter is obtained by taking the square-root of its variance. Since the m_L/m_S ratio depends only on the relative amplitudes of the L_3 and L_2 peaks and is independent of absolute amplitude scaling, the total relative uncertainty of the curve-fitting parameters was calculated as:

$$E_{uncertainty} = \frac{\sqrt{\sum_{p=1}^{n} \sigma_p^2}}{a_{\max}}$$

where σ_p^2 is the variance of pth curve-fitting parameter, n is the total number of freely varying curve-fitting parameters and $a_{\max}$ is the maximum amplitude of EMCD signal.

S 3.2. Random Error

Random error was determined taking the square root of the sum of squares of residual signal between the fit and the original EMCD signal divided by the maximum amplitude of the EMCD signal.

$$E_{random} = \frac{\sqrt{\frac{1}{n}\sum (f(X_k) - Y_k)^2}}{a_{\max}}$$

Where $f(X_k)$ is the fit function, Y_k is the original EMCD signal and n is the total number of data points in the EMCD signal.

S 3.3. Total Error

Total error was calculated by taking the quadrature of the two errors.

$$E_{total} = \sqrt{E_{uncertainty}^2 + E_{random}^2}$$

The curve fitting and calculation of error bars was carried out using scipy and numpy libraries in Python and the corresponding code is available in the data processing workflow file which is freely available at Zenodo[5].

1. Rusz, J. *et al*. Magnetic measurements with atomic-plane resolution. *Nat Commun* **7**, 12672 (2016).
2. Thersleff, T., Rusz, J., Hjörvarsson, B. & Leifer, K. Detection of magnetic circular dichroism with subnanometer convergent electron beams. *Phys Rev B* **94**, 134430 (2016).
3. EELS Atlas | EELS.info. https://eels.info/atlas.
4. Ali, H. *et al*. Single scan STEM-EMCD in 3-beam orientation using a quadruple aperture. *Ultramicroscopy* **251**, 113760 (2023).
5. Ali, H. *Atomic Plane Resolved STEM-EMCD*. https://doi.org/10.5281/zenodo.14827898 (2025).
